# Overexpression of RBBP6, Alone or Combined with Mutant TP53, Is Predictive of Poor Prognosis in Colon Cancer

**DOI:** 10.1371/journal.pone.0066524

**Published:** 2013-06-17

**Authors:** Jian Chen, Huamei Tang, Zehua Wu, Chongzhi Zhou, Tao Jiang, Yingming Xue, Guoyu Huang, Dongwang Yan, Zhihai Peng

**Affiliations:** 1 Department of General Surgery, Shanghai Jiaotong University Affiliated First People’s Hospital, Shanghai, People’s Republic of China; 2 Department of Pathology, Shanghai Jiaotong University Affiliated First People’s Hospital, Shanghai, People’s Republic of China; INRS, Canada

## Abstract

Retinoblastoma binding protein 6 (RBBP6) plays an important role in chaperone-mediated ubiquitination and interacts with TP53 in carcinogenesis. However, the clinicopathologic significance of RBBP6 expression in colon cancer is unknown; in particular, the prognostic value of RBBP6 combined with TP53 expression has not been explored. Therefore, quantitative real-time PCR and western blot analyses were performed to detect RBBP6 expression in colon cancer tissues. RBBP6 and TP53 expression were assessed by immunohistochemistry in a tissue microarray format, in which the primary colon cancer tissue was paired with noncancerous tissue. Tissue specimens were obtained from 203 patients. We found that RBBP6 was overexpressed in colon tumorous tissues and was significantly associated with clinical stage, depth of tumor invasion, lymph node metastasis (LNM), distant metastasis, and histologic grade. Further studies revealed that a corresponding correlation between RBBP6 overexpression and mutant TP53 was evident in colon cancer (r = 0.450; *P*<0.001). RBBP6 expression was an independent prognostic factor for overall survival (OS) and disease free survival (DFS). Interestingly, patients with tumors that had both RBBP6 overexpression and mutant TP53 protein accumulation relapsed and died within a significantly short period after surgery (*P*<0.001). Multivariate analysis showed that patients with LNM and patients with both RBBP6- and TP53-positive tumors had extremely poor OS (HR 6.75; 95% CI 2.63–17.35; *P*<0.001) and DFS (HR 8.08; 95% CI 2.80–23.30; *P*<0.001). These clinical findings indicate that the assessment of both RBBP6 and mutant TP53 expression will be helpful in predicting colon cancer prognosis.

## Introduction

As one of the most common cancers worldwide, colon cancer is a major cause of mortality. Surgical resection is the mainstay treatment for colon carcinoma. Tumor recurrence is the main factor for failure of colon cancer therapy following surgery. [1] Tumor node metastasis (TNM) staging is mainly used for risk assessment of colon cancer recurrence. [2] However, patients at the same tumor stage could have variable clinical outcomes. [3] Therefore, the discovery of novel markers for better prediction of post-surgery tumor recurrence is needed.

Cumulative clinical evidence showed that changes in cell-cycle regulator ubiquitination is associated with several human malignancies. [4] The high efficiency and exquisite selectivity of ubiquitination reactions are mediated by enzymes known as ubiquitin-protein ligases or E3s. [5] The protein encoded by the retinoblastoma binding protein 6 (*RBBP6*) gene, located on chromosome 16p11.2-p12, was suggested to possess E3-like activity. [6] RBBP6 strongly localises to chromosomes during mitosis and to nuclear speckles, whose overexpression could lead to cell cycle arrest, a common feature of tumorigenesis. [7] Up-regulation of *RBBP6* was strongly correlated with tumor progression in cervical and esophageal cancers, suggesting that *RBBP6* plays a crucial role in the malignant phenotype of human cancers. [8], [9] However, little is known about the clinical and pathological significance and prognostic value of *RBBP6* expression in colon cancer.

RBBP6 is one of the few proteins identified that has been shown to interact with TP53. [10] Through its RING finger-like domain, RBBP6 ubiquitinates TP53 by Mdm2, an E3 ubiquitin ligase. [11] In most human cancers, the tumor suppressor TP53 is mutated, leading to TP53 malfunction and acquired oncogenic activities. [12], [13] Mutations of TP53, which are considered to be able to bear ubiquitination, are thought to be involved in the pathogenesis of as many as 60% of colon cancers.[14]–[16] As the half-life of the wild-type TP53 protein is considerably short (5±20 min) and is consistent with its rapid turnover, it is usually undetectable by standard immunohistochemistry. Therefore, the detected TP53 protein was presumed to be the mutated TP53 protein. [11] Mutated genes are ideal targets for therapy, as shown in a recent study, which reported that regular aspirin consumption by patients diagnosed with colorectal cancer is associated with longer survival among patients with mutated-PIK3CA colorectal cancer but not among patients with wild-type PIK3CA cancer. [17] To date, the evidence for association of TP53 mutations with colon cancer prognosis was heterogeneous. [18] Moreover, the correlation between mutants TP53 and RBBP6, especially the prognostic value of their combined expression pattern, has not been analyzed in colon cancer.

In this study, we assessed the expression of RBBP6 and mutant TP53 in human colon cancer tissues, and their correlation with clinicopathologic features and patient survival. We also examined whether the combined expression of RBBP6 and mutant TP53 could serve as predictive markers for patient prognosis.

## Materials and Methods

### Human Tissue Specimens and Patient Information

Tissue specimens were obtained from 203 patients with colon cancer permitted operation by the General Surgery Department of Shanghai Jiao Tong University Affiliated First People’s Hospital between January 2001 and December 2003. Detailed information about the patient description is provided in the previous report. [19] There were 86 male and 117 female patients, with a median age of 68 years (range, 22–95 years) at the time of operation. The diagnoses were confirmed by at least 2 pathologists, and staging was determined in accordance with the American Joint Committee on Cancer (AJCC). The patients’ disease-free survival (DFS) and overall survival (OS) durations were defined as the period from initial surgery to clinically proven metastasis or recurrence and death, respectively. The median patient follow-up time was 61 months after surgery (range, 9–89 months). The study was approved by the Institutional Review Boards of Shanghai Jiaotong University and the affiliated Shanghai First People’s Hospital Medical Center. Written and informed consent was obtained from each patient before enrollment in the study.

### RNA Extraction and Quantitative Real-time PCR (qPCR)

Forty paired specimens of frozen tissues were used for qPCR analysis. Total RNA extraction kit (Qiagen, Germany) and cDNA synthesis kit (Promega, USA) were used following the manufacturers’ instructions. *RBBP6* gene was amplified using forward primer 5′-ctccccatacacttcctctcc-3′, and antisense primer 5′-ttcttttagtcgtcgctgctc-3′. qPCR was performed on a Mastercycler ep Realplex (Eppendorf) using the IQTM SYBR Green Supermix Kit (BIO-RAD) in accordance with the manufacturer’s protocol. Cycling conditions were as follows: initial denaturation (1 min at 95°C) followed by 25 cycles of denaturation for 1 min at 94°C, annealing for 1 min at 94°C, and elongation for 45 s at 72°C, with a final extension for 5 min at 72°C. Glyceraldehyde-3-phosphate dehydrogenase (GAPDH) was used as an internal control. The fold change 2-ΔΔCt of *RBBP6* expression was calculated using the formulas: RBBP6ΔCt = Avg.RBBP6_Ct – Avg. GAPDH_Ct and RBBPΔΔCt = RBBP6ΔCt_tumor – RBBP6ΔCt_non-tumor.

Univariate analysis showed that both increased postoperative recurrence and decreased OS were associated with pT stage, pN stage, M stage, AJCC stage, vascular invasion, tumor differentiation, and RBBP6 expression. RBBP6 overexpression combined with mutant TP53 protein accumulation is associated with markedly poorer OS (*P*<0.001) and DFS (*P*<0.001; Table 3).

### Western Blot Analysis

Tissue protein was extracted from colon tumor tissues and adjacent normal tissues of 4 patients by using the radio immunoprecipitation assay lysis buffer (50 mM Tris pH 7.4, 150 mM NaCl, 1% NP-40, 0.5% sodium deoxycholate and 0.1% sodium dodecyl sulphate), and protein concentration was determined using the BCA protein assay kit (Beyotime Biotechnology, Jiangsu, China). Equivalent amounts of protein was separated on 12% sodium dodecyl sulphate–polyacrylamide gels and then transferred onto polyvinylidene difluoride membranes. The membranes were blocked in 5% fat free milk with 0.1% Tween 20 for 1 h at room temperature, followed by incubation with primary antibody (1∶200 dilution for RBBP, 1∶500 for TP53, 1∶1000 for GAPDH; all purchased from Abcam, UK) and secondary antibodies conjugated to horseradish peroxidase. The proteins were detected by enhanced chemiluminescence (Pierce Biotechnology, Rockford, IL, USA) following the manufacturer’s instructions.

### Tissue Microarray (TMA) Construction and Immunohistochemistry

TMA slides were constructed as previously described. [1] Immunostaining was performed using the primary antibody against RBBP6 and TP53 (each 1∶100; Abcam, UK), and then incubated with the secondary antibody (Genetech, Shanghai, China). Tissue sections were counterstained with Mayer’s hematoxylin. Two researchers who were blinded to patient prognosis evaluated the slides independently. The staining intensity for RBBP6 was scored as 0 (no staining), 1 (mild staining), 2 (moderate staining), and 3 (intense staining). Staining area was scored as 0 (0%), 1 (1%–25%), 2 (26%–50%), 3 (51%–75%), and 4 (76%–100%) on the basis of the percentage of positively stained cells. [20] The final staining score, which is the sum of the intensity and extension scores, was divided into 3 groups as follows: 0–2, negative expression; 3–4, weak expression; and 5–6, strong expression. The TP53 index was divided into 2 groups: negative (less than 10% of cells with positive nuclei) and positive (more than 10% of cells with positive nuclei). [21].

### Statistical Analysis

The two-tailed χ^2^ test and Fisher exact test were used to determine the statistical significance of differences between the experimental groups. The correlation between RBBP6 and TP53 protein expression was calculated using Spearman’s test. The survival rates were calculated using the Kaplan-Meier method. A log-rank test was used to compare the survival curves. A Cox proportional hazards model was used to calculate univariate and multivariate hazard ratios for the variables. A *P*-value of less than 0.05 was considered statistically significant. All statistical analyses were carried out using the SPSS 19.0 statistical software package (SPSS Inc., Chicago, IL).

## Results

### Overexpression of RBBP6 in Colon Cancerous Tissues

**Figure 1:**
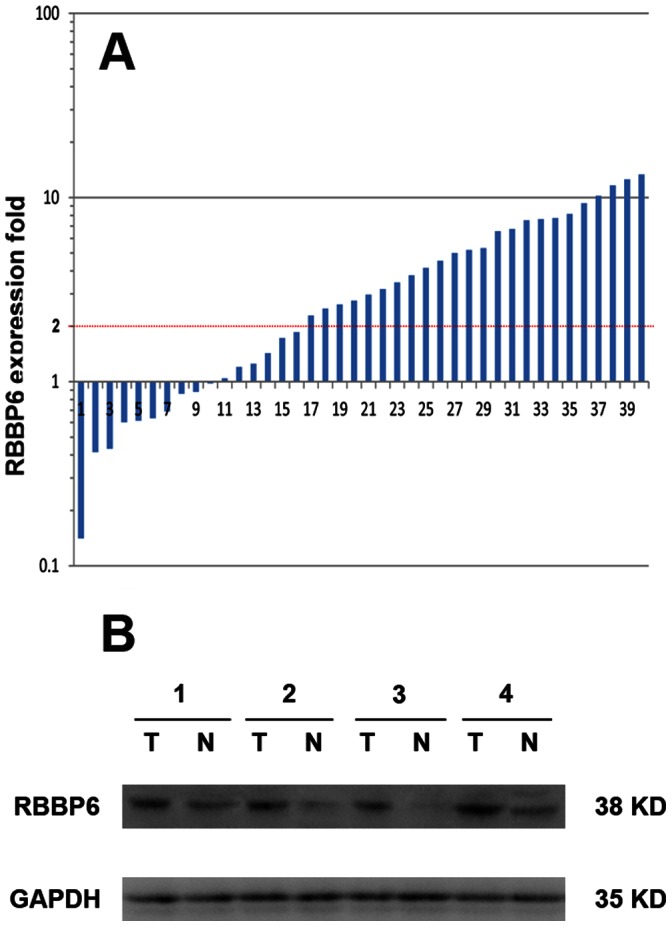
Expression of *RBBP6* in colon tumorous tissues and adjacent normal mucosa. **A.** Relative expression of *RBBP6* gene in a series of 40 matched colon cancerous tissue specimens compared with that in normal mucosa specimens. A logarithmic scale of 2− ^ΔΔCT^ was used to represent the fold change in quantitative real-time PCR detection. **B.** Western blotting analysis of RBBP6 protein expression in representative 4 paired colon tumor tissues. GAPDH was used as the loading control.

Among the 40 paired specimens subject to qPCR analysis, 24 (60%) colon cancers showed at least a 2-fold increase in RBBP6 mRNA levels compared with that of the adjacent non-cancerous tissues (Figure 1A). The RBBP6 mean relative quantification in the colon cancerous tissue group (4.88±0.76; 1.40–5.73) was significantly higher than that in the normal tissue group (3.49±0.43; 2.61–6.90; *P*<0.001). Of the 40 colon cancer samples, 4 showed strong up-regulation of RBBP6 mRNA expression that was determined by qPCR, and the RBBP6 protein expression in the 4 samples was evaluated by western blot analysis. All 4 samples showed higher RBBP6 protein levels than that of the adjacent noncancerous tissue (Figure 1B), suggesting that RBBP6 expression was elevated at both transcriptional and posttranscriptional levels.

### Association of RBBP6 and Mutant TP53 Expression with Clinicopathologic Parameters

**Figure 2:**
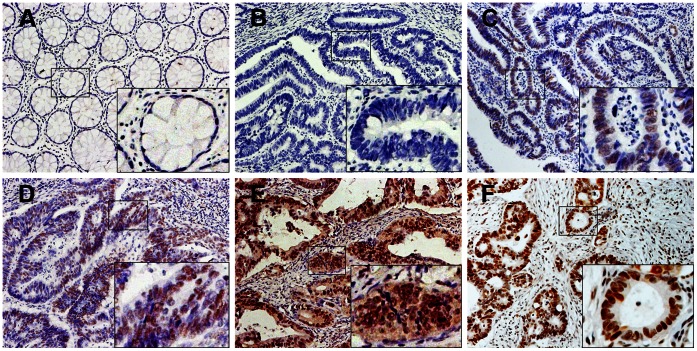
Immunohistochemical staining for RBBP6 expression in normal and colon cancer tissues. (A) Negative RBBP6 expression in normal colonic epithelium and (B) well-differentiated tumor. (C) Weak RBBP6 staining in a well-differentiated colon tumor. (D) Diffused, intense RBBP6 staining in moderately- and (E) poorly differentiated colon tumors. (F) Strong RBBP6 staining in a colon cancer lymph node metastasis sample. Original magnification ×200 (×400 for inset images).

**Figure 3:**
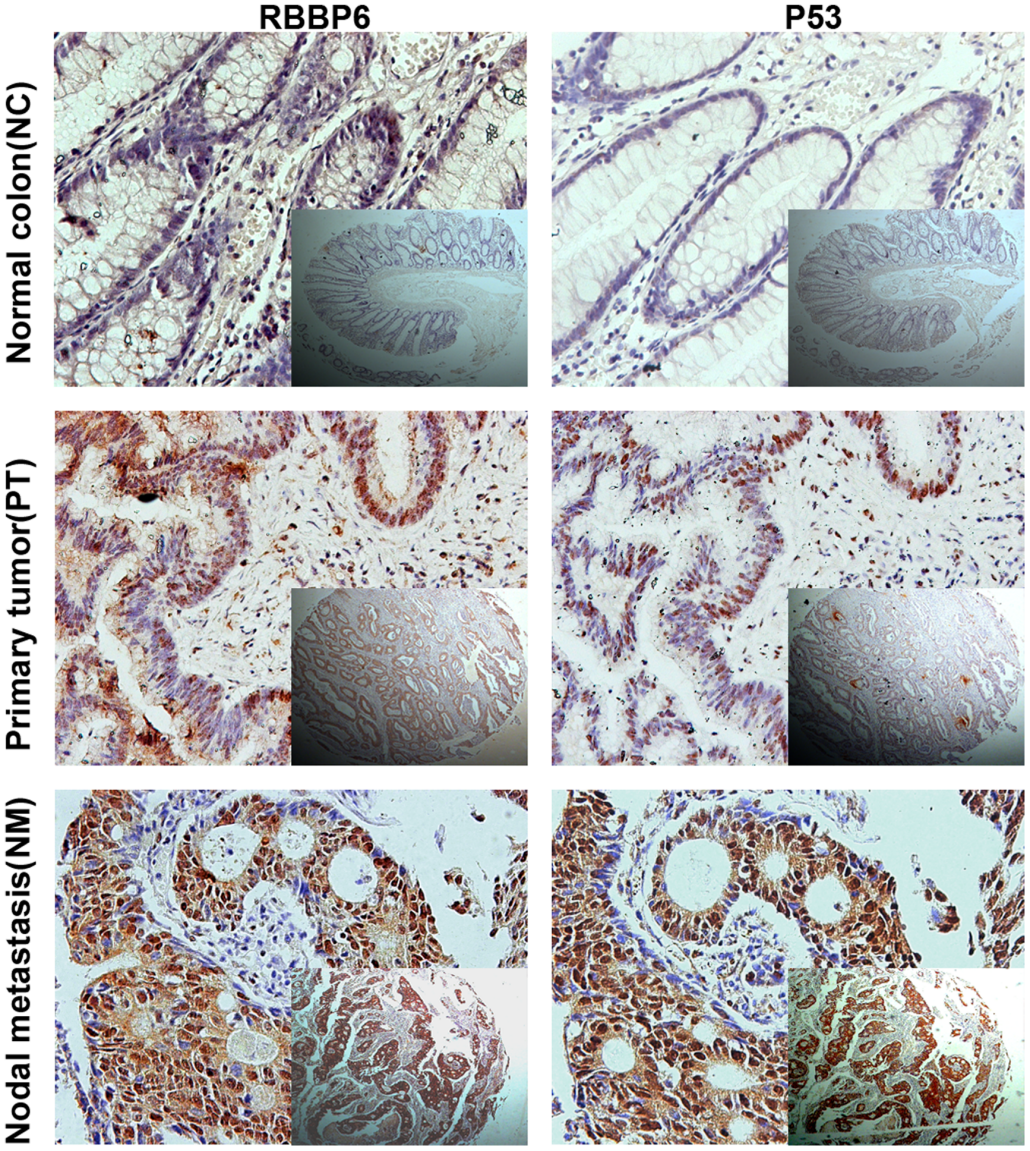
Expression of RBBP6 and **TP53.** Representative photographs of RBBP6 and TP53 expression in normal colon, colon tumor, and nodal metastasis specimens. RBBP6 expression was more frequently detected in specimens that stained positively for mutant TP53. Original magnification ×400 (×50 for inset images).

Of the 203 specimens on the paired TMA, 185 (91.1%) showed RBBP6 negative staining in normal mucosa. In contrast, RBBP6 was prominently expressed in colon cancerous tissue specimens, with strong staining in 56 (27.6%) samples, weak staining in 86 (42.4%) samples, and negative staining in 61 (30.0%) samples (Table 1). Of the 66 samples of available lymph node metastasis (LNM) specimens, 58 samples (87.9%) showed RBBP6 overexpression (Table 1). Positive staining was observed mainly in the nuclei of cancer cells (Figure 2). Associations of RBBP6 expression and clinicopathologic factors are summarized in Table 2. Increased RBBP6 expression was significantly associated with depth of tumor invasion (pT stage, *P = *0.005), LNM (pN stage, *P* = 0.002), distant metastasis (M stage, *P*<0.001), advanced AJCC (P<0.001) and differentiation (*P* = 0.007) (Table 2). RBBP6 expressed higher in the nodal metastasis than those in the primary tumor and normal tissue (Figure 2). Moreover, RBBP6 was more frequently detected in specimens that stained positively for mutant TP53 (Figure 3), and a statistical correlation between RBBP6 and TP53 (r = 0.450, *P*<0.001; Table 2) was observed. TP53 showed a positive association with pT stage (*P* = 0.005), pN stage (*P*<0.001), M stage (*P* = 0.006), advanced AJCC stage (*P*<0.001) and tumor differentiation (*P* = 0.009) (Table 2).

**Table 1:** RBBP6 and TP53 immunohistochemical staining for protein expression in normal colonic mucosa, cancerous tissue, and lymph node metastasis.

Tissue sample	n	Expression of RBBP6			*P* value	Expression of TP53		*P* value
		Negative(%)	Weak(%)	Strong(%)		Negative(%)	Positive(%)	
Normal mucosa	203	185(91.1)	18(8.9)	0(0)	<0.001*	201(99.0)	2(1.0)	<0.001*
Tumor	203	61(30.0)	86(42.4)	56(27.6)		97(47.8)	106(52.2)	
LNM	66	8(12.1)	11(16.7)	47(71.2)		10(15.2)	56(84.8)	

* *P*-value is based on chi-square test; LNM: Lymph node metastasis.

**Table 2:** Association between clinicopathologic features and RBBP6 or TP53 protein expression.

	Expression of RBBP6			*P* value	Expression of TP53		*P* value
	Negative(n = 61)	Weak(n = 86)	Strong(n = 56)		Negative(n = 97)	Positive(n = 106)	
Age							
<65	21	37	23	0.564	36	45	0.670
≥65	40	49	33		61	61	
Gender							
Male	32	30	24	0.111	38	48	0.397
Female	29	56	32		59	58	
Location							
Right	24	37	23	0.732	42	42	0.746
Transverse	8	6	5		10	9	
left	29	43	28		45	55	
pT stage							
pT1	5	3	0	0.005 *	2	6	0.005*
pT2	14	5	4		17	6	
pT3	20	34	22		41	35	
pT4	22	44	30		37	59	
pN stage							
pN0	37	48	23	0.002*	64	44	<0.001*
pN1	10	27	14		27	34	
pN2	4	11	19		6	28	
M stage							
M0	60	82	43	<0.001*	94	91	0.006
M1	1	4	13		3	15	
AJCC stage							
I	15	6	3	<0.001*	16	8	<0.001*
II	21	41	19		48	33	
III	24	35	21		30	50	
IV	1	4	13		3	15	
Vessel invasion							
No	59	80	50	0.312	94	95	0.052
Yes	2	6	6		3	11	
Differentiation							
Well	39	42	18	0.007*	58	41	0.009*
Moderate	17	33	24		29	45	
poor	5	11	14		10	20	
TP53							
Negative	48	38	11	<0.001**			
Positive	13	48	45				

* *P*<0.05 indicates a significant association among the variables.

** The significant difference of correlation between RBBP6 and TP53 based on Spearman test.

### Overexpression of RBBP6 alone or Combined with Mutant TP53 Predicts Poor Prognosis

Of the 195 patients, 78 (40%) patients who underwent curative operations experienced disease relapse. Patients with RBBP6-positive tumors promptly showed metastases or local recurrence than those with RBBP6-negative tumors (weak: 40/46, 46.5%; strong: 33/48, 68.8%; negative: 5/61, 8.2%; *P*<0.001).

**Figure 4:**
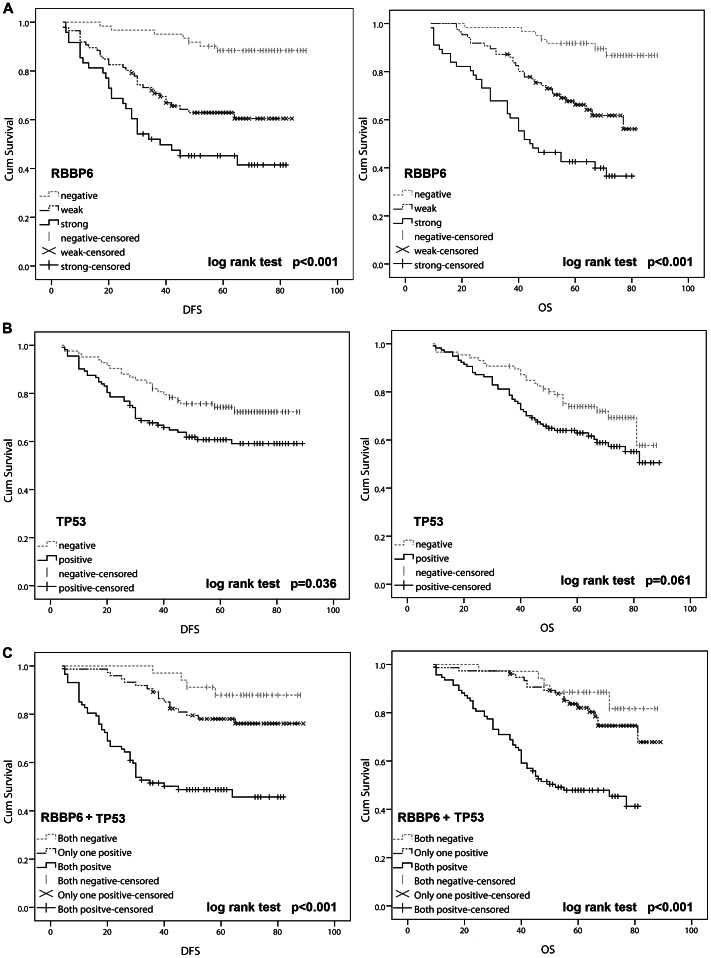
Kaplan-Meier analyses with a log rank test of survival. **A.** Disease-free survival (DFS) and overall survival (OS) of patients in relation with RBBP6 expression levels that were determined by immunohistochemical staining. **B.** DFS was significantly better in patients with TP53-negative tumors than in those with TP53-positive tumors (*P = *0.036). **C.** DFS and OS were significantly lower in patients with RBBP6− and TP53-positive tumors than in those with RBBP6− and TP53-negative tumors (*P*<0.001 for both).

The Kaplan-Meier plot showed that patients showing RBBP6 overexpression had a poor OS and DFS than patients with RBBP6-negative tumors (log rank, *P*<0.001; Figure 4A). On the other hand, mutant TP53 had no relation with OS but was significantly associated with DFS (*P*<0.001; Figure 4B). Furthermore, with regard to concomitant expression of RBBP6 and mutant TP53 proteins, we divided the specimens into 3 groups: group 1, tumors exhibiting no expression of RBBP6 and mutant TP53 (RBBP6−/TP53−, 48 specimens); group 2, tumors with abnormal expression of only 1 protein (RBBP6+/TP53−, or RBBP6−/TP53+, 62 specimens); and group 3, tumors with abnormal expression of both proteins (RBBP6+/TP53+, 49 specimens). Notably, there was a trend toward a better OS and DFS in the patient group with RBBP6− and TP53-negative tumors than that in the patient group with RBBP6− and TP53-positive tumors (*P*<0.001). Interestingly, the DFS and OS curves for patients with only RBBP6− or TP53-positive tumors were relatively close to those with tumors that were negative for both RBBP6 and TP53, but were dramatically discrepant from those of the RBBP6− and TP53-positive group (Figure 4C).

**Table 3:** Univariate and multivariate Cox proportional hazard models for overall survival (OS) and disease-free survival (DFS).

Variable	OS				DFS			
	Univariate		Multivariate		Univariate		Multivariate	
	HR (95% CI)	*P*-value	HR (95% CI)	*P-*value	HR (95% CI)	*P*-value	HR (95% CI)	*P*-value
RBBP6/TP53								
Both negative	1		1		1		1	
One positive	3.27 (1.08, 9.85)	0.036	2.55 (0.93, 6.95)	0.068	2.97 (0.98, 9.03)	0.055	3.45(1.15,10.40)	0.028*
Both positive	12.58(4.53,34.95)	<0.001*	6.75(2.63,17.35)	<0.001*	11.45(4.11,31.90)	<0.001*	8.08(2.80,23.30)	<0.001*
RBBP6								
Negative	1		1		1		1	
Weak	4.23 (1.86, 9.60)	0.001*	2.79(1.19,6.58)	0.019*	4.17(1.84,9.46)	0.001*	3.022(1.40,7.78)	0.006*
Strong	9.08(4.02,20.55)	<0.001*	2.94(1.16,7.43)	0.023*	7.43(3.23,17.12)	<0.001*	3.302(1.18,7.77)	0.043*
TP53								
Negative	1				1			
Positive	1.58(0.97,2.56)	0.064			1.71(1.03,2.86)	0.040*		
Age								
<65	1				1			
≥65	0.96(0.61,1.53)	0.875			1.06(0.65,1.75)	0.808		
Gender								
Male	1				1			
Female	1.34(0.84,2.16)	0.222			1.22(0.75,2.00)	0.427		
Location								
Right	1				1			
Transverse	0.96(0.42,2.19)	0.92			0.86(0.33,2.25)	0.758		
left	1.06 (0.64,1.76)	0.81			1.14(0.49,2.63)	0.768		
pT stage								
pT1	0.36(0.87,1.46)	0.15	0.15(0.31,0.74)	0.020*	0.38(0.93,1.58)	0.184	0.17(0.34,0.81)	0.027*
pT2	0.11(0.26,1.46)	0.002*	0.21(0.01,3.30)	0.264	0.13(0.30,0.52)	0.004*	0.22(0.02,2.89)	0.253
pT3	0.38(0.20,0.59)	<0.001*	0.37(0.21,0.664)	0.001*	0.36(0.20,0.63)	<0.001*	0.35(0.19,0.64)	0.001*
pT4	1		1		1		1	
pN stage								
pN0	1		1		1		1	
pN1	4.02 (2.18, 7.43)	<0.001*	3.44(0.67,17.72)	<0.001*	3.43(1.81,6.52)	<0.001*	1.29(0.17,9.77)	0.005*
pN2	14.07 (7.54, 26.27)	<0.001*	5.74(1.07,30.69)	<0.001*	14.18(7.48,26.89)	<0.001*	3.72(0.47,30.12)	<0.001*
AJCC stage								
I	1				1			
II	2.08 (0.47, 9.21)	0.34			2.00(0.45,8.7)	0.361		
III	9.51 (2.29, 39.50)	0.002*			9.05(2.19,37.50)	0.002*		
IV	72.12(16.17,321.61)	<0.001*			42.10(8.86,199,46)	<0.001*		
M stage								
M0	1				1		1	
M1	14.74(8.15,26.67)	<0.001*			9.02(4.32,18.86)	<0.001*	3.36(1.54,7.34)	0.002*
Vessel invasion								
No	1				1			
Yes	4.68 (2.545, 8.60)	<0.001*			5.16(2.74,9.74)	<0.001*		
Differentiation								
Well	1		1		1			
Moderate	2.368 (1.34, 4.18)	0.003*	1.96(1.08,3.57)	0.028*	2.34(1.31,4.19)	0.004*		
Poor	7.50 (4.11, 13.69)	<0.001*	3.61(1.74,7.49)	<0.001*	6.36(3.35,12.09)	<0.001*		

HR: Hazard ratio; CI: Confidence interval.

* *P*<0.05 indicated that 95% CI of HR was not including 1.

Multivariate analysis was performed using the Cox proportional hazards model for all of the significant variables in the univariate analysis. The results demonstrated that positive tumor RBBP6 expression was a significant independent prognostic factor for increased disease recurrence and decreased survival (Table 3). Additionally, although mutant TP53 alone was not a prognostic indicator, expression of both RBBP6 and mutant TP53 was found to be a significant independent prognostic factor for OS (HR 6.75; 95% CI, 2.63–17.35; *P*<0.001) and DFS (HR 8.08; 95% CI, 2.80–23.30; *P*<0.001).

## Discussion

To the best of our knowledge, this is the first study to report that both transcriptional and posttranscriptional RBBP6 levels are elevated in colon cancer. We found that RBBP6 expression was significantly associated with advanced cancer biology, which was indicated by invasion depth, LNM, and distant metastasis. These strong associations suggest that RBBP6 overexpression promotes tumor invasion and metastasis and that RBBP6 could possibly be used as a biomarker for a more aggressive phenotype of colon cancer.

It is now apparent that the deregulation of ubiquitin pathways results in many types of tumors. [22] RBBP6 has been identified as a putative E3 ubiquitin ligase because of the presence of a RING finger domain. [8] Several major types of E3 enzymes (RING/U-box families) have been linked to the development of cancer. [23] The human RBBP6 contains domains that are known to interact with TP53 and its function as ubiquitin might cause the deregulation of TP53, which would then lead to carcinogenesis. [6] However, the correlation between RBBP6 and mutant TP53 has not been analyzed in human colon cancer.

*TP53* is a major gene involved in the determination of proliferation or growth arrest at the cellular level. [24], [25] Mutant *TP53* occurs in nearly half of all cancer cases and may be a promising target for pharmacological reactivation. [26], [27] Mutant *TP53* contributes to development of cancer not only through loss of activity but also through gain of specific “mutant functions.” [28] Additionally, unlike wild-type TP53, which under normal conditions has a short half-life when it is targeted by Mdm2 for degradation, mutant TP53 proteins are outside this negative feedback loop and have an increased half-life, thus resulting in the emergence of gain-of function phenotypes. [29], [30] With a prolonged half-life, the mutant TP53 protein can therefore be detected through immunohistochemistry. [31] In this study, RBBP6 and mutant TP53 protein were overexpressed in colon cancer tissues and found to be positively correlated. The mechanisms for the association of RBBP6 up-regulation with mutant TP53 are poorly understood. Our finding provides some insight into this apparent conflict. We found that RBBP6 overexpression induces tumorigenesis through ubiquitination of TP53 by Mdm2; however, TP53 is the most commonly mutated gene known in human cancer. [32] It was assumed that mutant TP53 is more stable, perhaps because its altered conformation makes the protein less susceptible to degradation. [33] So it is possible that up-regulation of RBBP6 fails to ubiquitinate mutated TP53 genes. Mutant TP53 might also activate the *RBBP6* gene and promote colon tumorigenesis due to the loss of its anti-carcinoma effect. However, further studies are needed to elucidate the molecular mechanisms of *RBBP6* and *TP53* genes in the progression of colon cancer.

Although the understanding of metastatic process has greatly evolved, mechanisms involved in colon cancer metastasis are not fully understood. The results of the present study showed that positive RBBP6 staining was considerably higher in metastatic colon cancer cells within lymph nodes than in the paired primary tumors. RBBP6 expression was associated with an increased risk of metastasis/local recurrence and was strongly linked to poor survival outcomes. These data indicate that increased RBBP6 expression correlates with invasive behavior and metastatic processes of colon cancer. Multivariate analysis shows that RBBP6 expression alone or combined with mutant TP53 expression appeared to be an independent prognostic factor for OS and DFS in colon cancer. As patients with tumors that had both PIK3CA mutation and PTGS2 expression displayed the strongest therapeutic effect of aspirin [17], we proposed that data on RBBP6 and mutant TP53 expression in tumors could be used to design optimal, individualized treatment and to help identify patients who may or may not benefit from close monitoring after surgery. Additional research would be required to confirm our findings. Owing to the heterogeneous nature of patient population, the AJCC stage was excluded from the final multivariate Cox proportional hazard model. Although limitations include the small number of patients with relatively short follow-up time, our results provide the first evidence that RBBP6 could be used as a novel biomarker for improved outcome after colectomy in patients with colon cancer. Most of all, the mutant *TP53*, a gene significantly correlated with colon cancer, although not predictive of cancer prognosis by itself, could be of strong predictive value when evaluated with RBBP6 expression.

In summary, this study provided critical insight into the role of the *RBBP6* gene in the progression of colon cancer. The frequent up-regulation of RBBP6 expression in human colon cancer highlights its potential as a novel therapeutic target for this cancer. The findings reported here also indicate that RBBP6 overexpression in combination with mutant TP53 protein accumulation was associated with recurrent cancer and a poor survival rate. These findings might be helpful in designing future studies to understand the molecular development of colon cancer. The potential clinical value of RBBP6 alone or in combination with mutant TP53 as a novel biomarker in colon cancer should be investigated using randomized controlled studies.
